# Corrigendum to: C-reactive protein is associated with the development of tongue squamous cell carcinoma

**DOI:** 10.3724/abbs.2023284

**Published:** 2023-12-20

**Authors:** Jianxin Du, Wei Hu, Chengzhe Yang, Yegang Wang, Xiaoying Wang, Pishan Yang

*Acta Biochimica et Biophysica Sinica* 2018, 50(3): 238‒245.

https://doi.org/10.1093/abbs/gmy004

In the original version of this manuscript, an error was found in Figure 6. The correct figure is as follows. The authors apologize for the error.

**Figure:**
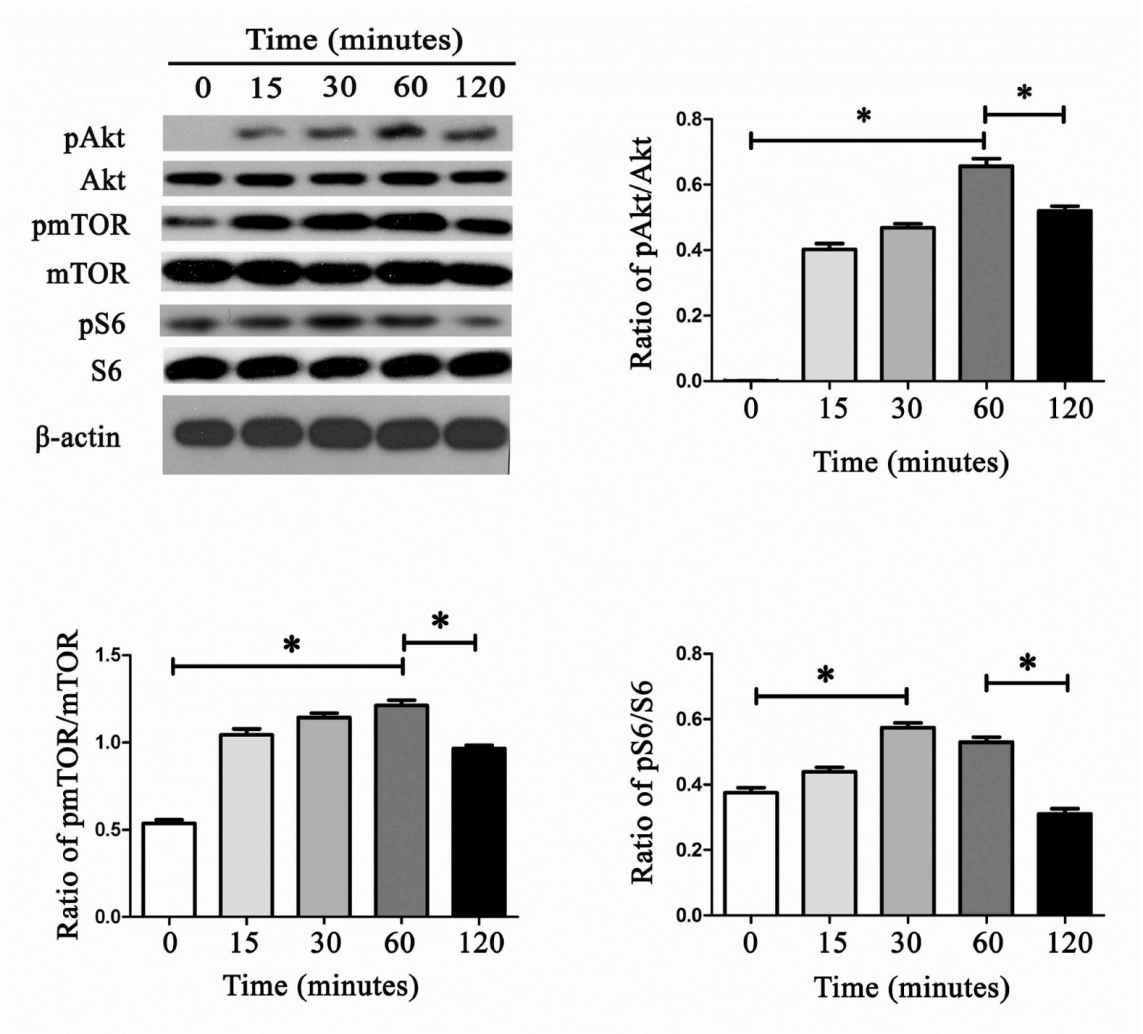
Figure 6 CRP treatment could remarkably induce Akt, mTOR and S6 phosphorylation With 10 μg/mL CRP stimulation, the expression levels of pAkt, pmTOR for up to 60 min and that of pS6 for up to 30 min were significantly up-regulated. When the treatment time was extended to 120 min, the protein expression levels of pAkt, pmTOR and pS6 were down-regulated. Data are presented as the mean±SEM, *P <0.05.

